# Characterization of γ′ Precipitates in Cast Ni-Based Superalloy and Their Behaviour at High-Homologous Temperatures Studied by TEM and in Situ XRD

**DOI:** 10.3390/ma13102397

**Published:** 2020-05-22

**Authors:** Łukasz Rakoczy, Ondrej Milkovič, Bogdan Rutkowski, Rafał Cygan, Małgorzata Grudzień-Rakoczy, František Kromka, Anna Zielińska-Lipiec

**Affiliations:** 1 Faculty of Metals Engineering and Industrial Computer Science, AGH University of Science and Technology, Mickiewicza 30, 30-059 Cracow, Poland; rutkowsk@agh.edu.pl (B.R.); alipiec@agh.edu.pl (A.Z.-L.); 2 Institute of Materials Research, Slovak Academy of Sciences, Watsonova 47, 040 01 Košice, Slovakia; omilkovic@saske.sk (O.M.); fkromka@saske.sk (F.K.); 3 Institute of Experimental Physics, Slovak Academy of Sciences, Watsonova 47, 040 01 Košice, Slovakia; 4 Consolidated Precision Products, Investment Casting Division, Hetmańska 120, 35-078 Rzeszów, Poland; Rafal.Cygan@cppcorp.com; 5 Lukasiewicz Research Network-Krakow Institute of Technology, Zakopianska 73, 30-418 Krakow, Poland; malgorzata.grudzien@kit.lukasiewicz.gov.pl; 6 Faculty of Materials, Metallurgy and Recycling, Technical University of Košice, Letná 9, 042 00 Košice, Slovakia

**Keywords:** misfit, in situ XRD, lattice parameter, STEM, gamma prime, dissolution

## Abstract

In situ X-ray diffraction and transmission electron microscopy has been used to investigate René 108 Ni-based superalloy after short-term annealing at high-homologous temperatures. Current work is focused on characterisation of γ′ precipitates, their volume fraction, evolution of the lattice parameter of γ and γ′ phases and misfit parameter of γ′ in the matrix. Material in the initial condition is characterised by a high-volume fraction (over 63%) of γ′ precipitates. Irregular distribution of alloying elements was observed. Matrix channels were strongly enriched in Cr, Co, W and Mo, whereas precipitates contain large amount of Al, Ti, Ta and Hf. Exposure to high-homologous temperatures in the range 1100–1250 °C led to the dissolution of the precipitates, which influenced the change of lattice parameter of both γ and γ′ phases. The lattice parameter of the matrix continuously grew during holding at high temperatures, which had a dominant influence on the more negative misfit coefficient.

## 1. Introduction

Nickel-based superalloys, due to their ability to keep high strength, very good hot corrosion and oxidation resistance during long-term service, are commonly used in high-homologous temperature applications. The binary Ni-Al phase diagram includes various ordered phases, characterised by the high ordering enthalpy (e.g., NiAl, NiAl_3_, Ni_2_Al_3_, NiAl_9_ and, the most important, Ni_3_Al) [1,2,3,4,5,6]. The γ matrix of superalloys is strengthened by coherent γ′ phase, possessing an ordered L1_2_ structure. Taking into account operation at high temperature, the volume fraction, misfit, coarsening rate and antiphase boundary energy (related to the chemical composition) of the γ′ precipitates plays an important role [7,8,9]. Ordering of the γ′ phase is dependent on the strictly defined location of Al and Ni atoms in its crystal lattice, which influences further behaviour during high temperature exposure and deformation. Ni_3_Al is very rigid and impedes dislocation movement from the γ matrix, ensuring required strength under operation conditions. High degree of directional, covalent bonding (molecular bonds) leads to the accurate stoichiometric relation between the number of Al and Ni atoms in the unit cell. In Ni_3_Al crystal structure, Ni-Al, rather than Ni-Ni and Al-Al, chemical bonds are favoured [10]. Distance between Ni and Al atoms is calculated as $\sqrt{2}a$, therefore Al is preferentially substituted by large atoms. The alloying elements Ti, Ta, Nb and Pt are γ′ formers and dissolve in the γ′ phase. The second group is γ stabilisers, namely Co, Cr, Mo, Re, Ru and Ir [11]. The lattice misfit parameter and the volume fraction of the γ′ phase in the matrix are among the most important microstructural features, which have a strong effect on the dislocation density and evolution rate of dislocation networks, and consequently the creep life of superalloys [12,13,14,15,16,17,18]. The misfit parameter is usually defined as $\delta =\frac{2\left(a_{\gamma^{\prime }}-a_{\gamma }\right)}{a_{\gamma^{\prime }}+a_{\gamma }}$, where a_γ_ and a_γ′_ are lattice parameter of the γ and γʹ phases, respectively [19,20].

Over the years, considerable effort has been expended in attempts to correlate γ/γ′ lattice misfit to the strength of Ni-base superalloys. Maniar [21] observed that a misfit close to zero considerably improves high temperature strength. Equally important was the content of γ′ formers and resultant volume fraction of γ′. At similar misfit value, the stress rupture life could be further improved by increased volume fraction of γ′. The appropriate design of only the misfit increased the rupture life of a model superalloy at 760 °C/379 MPa almost twice, whereas optimisation of both, misfit and volume fraction of γ′- by more than four times. The influence of the misfit parameter, induced by addition of various contents of alloying elements, on the strength of Ni-15Cr-Al-Ti-Mo alloys at five temperatures between 25 and 800 °C led to the conclusion that relationship between the δ parameter and the strength of a superalloy (after accounting for the fraction of γ′ phase) is linear [22]. At relatively low temperatures, in the condition of dislocation creep, a relatively high misfit strain in the matrix increases strengthening. At high homologous temperatures (T > 0.6 T_L_) a lower lattice misfit reduces γ/γ′ interface energy, which maximises phase stability and decreases coarsening kinetics. Since the distance between the γ′ precipitates is usually less than 0.1 μm, the γ matrix never attains its strain-free (equilibrium) lattice parameter. So, the misfit measured for the standard heat treatment condition (solution + ageing), with a fully coherent γ′ phase, is not the equilibrium value, but a constrained value for the superalloy [23,24,25]. The start temperature of γ′ dissolution (T_sd_) for Inconel 738, determined by Zla [26] by differential thermal analysis, was reported to be between 914 and 957 °C, depending on the heating rate, and for Inconel 792, Strunz [27] showed T_sd_ to be below 900 °C. In PM Astroloy, the start of dissolution occurred between 800 and 850 °C [28]. Sponseller [29] calculated γ′ solvus temperature (T_s_) for some superalloys to be between 1189 °C (equiaxed René 95) and 1307 °C (CMSX-2), and so the interval was also relatively wide. Critical temperatures are not constant and strongly dependent on the heating rate, as confirmed by Soucail [28]. The most outstanding feature concerns the large deviation for the solvus temperature. Under equilibrium conditions, it was 1140 °C, whereas heating at 300 °C s^−1^ caused a displacement up to 1265 °C.

The aim of this investigation was to characterise the stereological parameters and the chemical composition of γ′ precipitates in René 108 in the initial condition, and also show their behaviour during dissolution at high-homologous temperatures, by studying the evolution of lattice parameter of γ and γ′ and the resultant misfit parameter. These changes are especially important for several reasons. The first was the selection of the solution heat treatment temperature and time, in order to homogenise the microstructure as much as possible. The second, as several reports [30,31,32] had indicated, that superalloys strengthened by a high-volume fraction of γ′ precipitates are strongly susceptible to liquation cracking during welding. Some investigations [33,34,35] indicate that high temperature preheat can overcome this problem, so it is important to study the kinetics of the dissolution process to prevent this negative result. The next factor was the selection of post-weld heat treatment parameters, which is mandatory to achieve the required mechanical properties of the component. The detailed kinetics of γ′ dissolution and change of misfit with temperature and time are presented in the literature only for a few alloys, but data about René 108 are missing. This work was carried out in order to complete this lack of useful information.

## 2. Experimental Procedure

The lost-wax castings of René 108 nickel-based superalloy were heat treated in vacuum with the following parameters: (1) solution: 1200 °C, 2 h; (2) ageing: 900 °C, 8 h. The chemical composition, determined by optical emission spectroscopy (OES), was (wt %): Cr: 11.36, Co: 8.84, W: 8.19, Al: 6.36, Ta: 3.55, Hf: 1.5, Ti: 0.75, Mo: 0.45, C: 0.07, B: 0.015, Zr: 0.0125, Ni: Bal. The specimens for scanning electron microscopy (SEM) observations, cut from the casting, had dimensions of 30 × 30 × 30 mm. Afterwards, they were ground, polished on diamond suspensions and finally electrochemically etched in 10% oxalic acid. Observations were carried out using a Phenom XL microscope (Phenom-World, Eindhoven, Netherlands) (backscattered electron imaging), with an accelerating voltage of 20 kV. Image analysis of the γ′ precipitates in dendritic regions was carried out by ImageJ commercial software (developed at the National Institutes and the Laboratory for Optical and Computational Instrumentation, University of Wisconsin, Madison, WI, USA) in 10 regions at ×20,000 magnification (area-179.8 μm^2^). Each image was subjected to binarisation and a despeckle filter, which removed noise without blurring edges. The area (A) and perimeter (P) of each precipitate in a range of 0.06–5.0 μm^2^ were measured. Based on these values, the equivalent length of the square’s side of precipitates (square root of precipitates’ area) and shape factor $(\xi =\frac{4\pi A}{P^{2}}$) were determined. Specimens for transmission electron microscopy were firstly ground mechanically to a thickness of about 0.05 mm, and then 3 mm discs were punched and dimpled on each side. The last step was thinning by Ar^+^ ion beam (PIPS of Gatan). The probe Cs-corrected FEI Titan^3^ G2 60-300 with a ChemiSTEM system (Thermo Fisher Scientific) was used to obtain nanostructure and chemical composition of the γ matrix and γ′ precipitates. Based on TEM images, the stereological parameters of nanoprecipitates of γ′ in the thin channel matrix were determined. High resolution images were subjected to fast Fourier transformation (FFT), to reveal the position of diffraction peaks. Segregation coefficient of alloying elements between matrix and γ′ was calculated according to the equation $k_{\gamma \prime }^{i}=\frac{C_{\gamma \prime }^{i}}{C_{\gamma }^{i}}$ (concentration of alloying element in the γ′ precipitates divided by concentration in channels of matrix). X-ray diffraction experiments were carried out on a Philips X’Pert Pro MRD diffractometer, (Philips, Amsterdam, Netherlands) using Cu K_α_ radiation in Bragg-Brentano geometry. High temperature measurements were realised on samples placed on the platinum strip in the heating chamber (Anton Paar HTK16). The samples were wire cut from the blade holder part into rods with a diameter of 6 mm and subsequently sliced and polished into discs with a thickness of 30 μm. Specimens were heated at a rate 100 °C/min, held for 30 s in order to homogenise the temperature in the sample volume, and then the diffraction measurement was started. The in situ study was realised for four temperatures: 1100 °C, 1150 °C, 1200 °C and 1250 °C. To record the high temperature time-resolved processes, samples were measured repeatedly at a narrow interval of 2ϴ between 41.5° and 46.5° to capture the (111) peak from the superalloy and the (200) peak from the Pt plate. Collected data were fitted by well-known procedures using the pseudo-Voigt profile function to determine the structure parameters. The angles were read off from the positions of the peaks on a diffractogram and the interplanar spacings d_khl_ were calculated using the Bragg-Wulff equation (Equation (1)). Based on the computed lattice parameter of matrix a_γ_ and precipitates a_γ′_ (Equation (2)), the misfit parameter was determined. The thermodynamic simulations using Thermo-Calc software (version 2020a, Thermo-Calc software, Solna, Sweden) (database TCNI6:Ni-Alloys) were carried out, in order to calculate the maximum solubility of γ′ formers in the Ni matrix with an increase of the test temperature.

(1) $$d_{hkl}=\frac{\lambda }{2sin\theta }$$

(2) $$a=\frac{\lambda \;\sqrt{h^{2}+k^{2}+l^{2}}}{2sin\theta }$$

## 3. Results and Discussion

### 3.1. Characterisation of γ and γ′ Phases Structure in the Initial Condition

**Figure 1:**
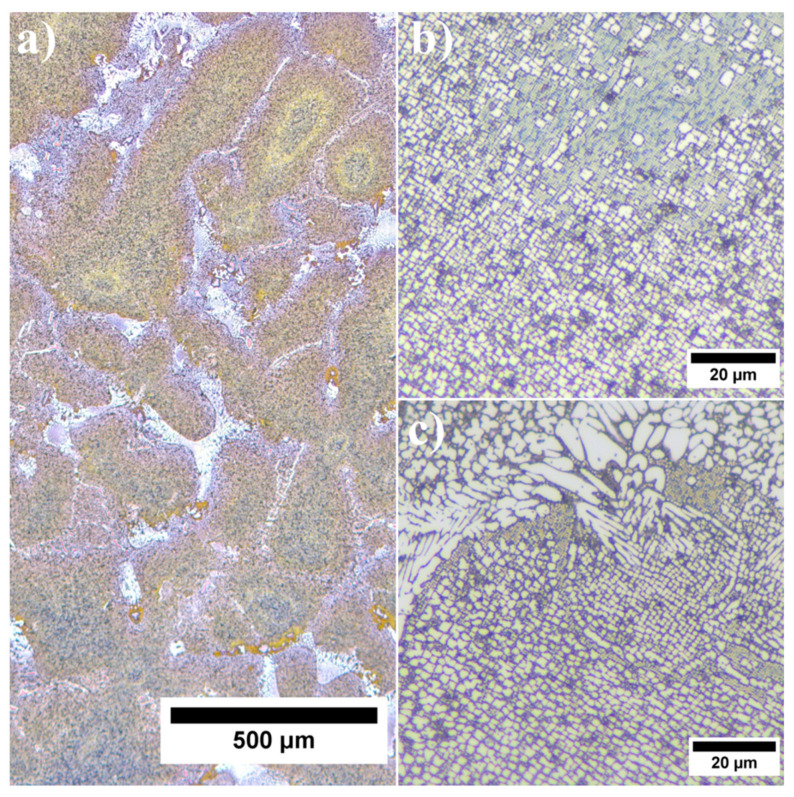
Inhomogeneous microstructure of René 108 superalloy: (**a**) dendritic structure; (**b**) precipitates in dendritic regions; (**c**) precipitates in interdendritic spaces.

In the initial condition René 108 superalloy is characterised by a dendritic structure, typical for castings (Figure 1a). Despite the heat treatment (solution + ageing), the structure was still very heterogeneous, and revealed differences in size and morphology of precipitates. Dendritic regions consisted of γ and γ′ precipitates, whereas in the interdendritic spaces there were additionally carbides and eutectic γ-γ′ islands (Figure 1b,c). 

**Figure 2:**
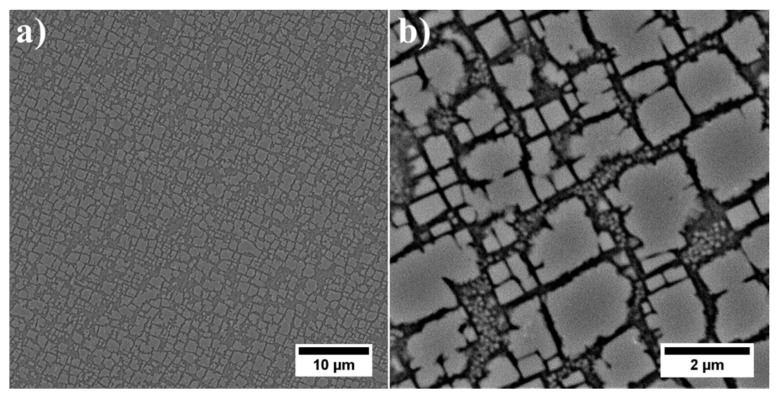
Microstructure of γ′ precipitates in dendritic region: (**a**) distribution of large secondary γ′ precipitates; (**b**) tertiary γ′ precipitates in channels of γ matrix.

Dendritic regions had a relatively high-volume fraction of γ′ precipitates, characterised by a typical cubic or near cubic-shaped morphology (Figure 2a). The edges of the largest secondary γ′ were quite irregular, due to an increasing degree of ageing. They were formed via the coagulation and coalescence of γ′ precipitates during heat treatment. The misfit stresses at γ/γ′ interfaces favoured nucleation of precipitates in the stress field in the corners. For the near-cubic precipitates the coagulation rate was extremely low, and their morphology did not evolve strongly during heat treatment. Within the γ matrix channels, numerous smaller tertiary γ′ precipitates were also present (Figure 2b). Such morphology and location indicated their formation during cooling after ageing. In the wider channels they probably nucleated on the non-completely dissolved (residual secondary γ′) precipitates, whereas in thin channels, the smallest γ′ formed. Their morphology, based on TEM images, is discussed later.

**Figure 3:**
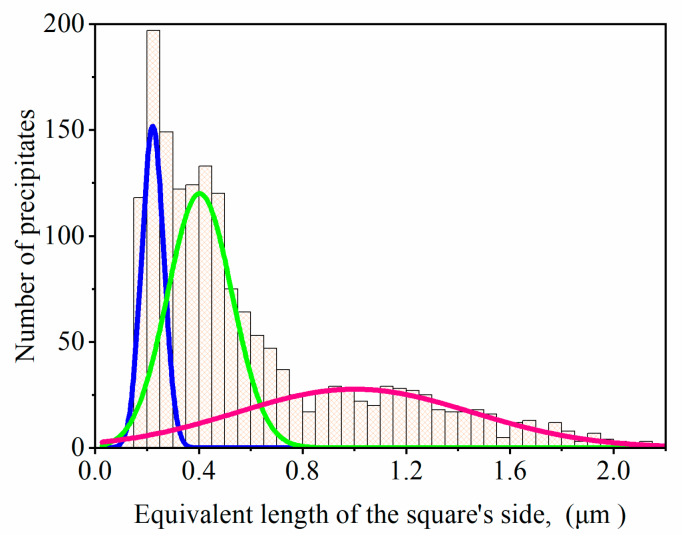
Histogram of equivalent length of the square’s side of γ′ precipitates.

Stereological analysis for γ′ precipitates was performed for 10 locations in a dendritic region. The mean area of precipitates was 0.52 μm^2^ (±0.82 μm^2^) and their perimeter 3.51 μm (±3.42 μm). The size of precipitates expressed as equivalent length of the square’s side was 0.61 μm (±0.44 μm). All the calculated values were presented as a histogram with fitted curves (Figure 3). Three classes of precipitates are shown, large secondary γ′ (the mean size 1.0 μm-pink curve), fine secondary γ′ with near-cubic morphology (the mean size 0.4 μm-green curve) and also the largest amount of tertiary γ′ precipitates in wide channels (the mean size 0.22 μm-blue curve). The stereological parameters of tertiary γ′ nanoprecipitates were investigated on TEM images. In accordance with the shape factor equation $(\xi =\frac{4\pi A}{P^{2}}$), the precipitates with a perfectly cubic shape have a coefficient of ζ 0.785, while the ideal sphere exhibits ζ of 1.0. The shape factor of γ′ precipitates was 0.51, indicating a much more complex geometry than a perfect cube. The standard deviations of the parameters were relatively high, due to the large diversity in particle area, which is typical for many equiaxed superalloys, in contrast to the single crystal variant [1,2]. It is related to the various chemical compositions, period of formation and also interfacial stresses.

**Figure 4:**
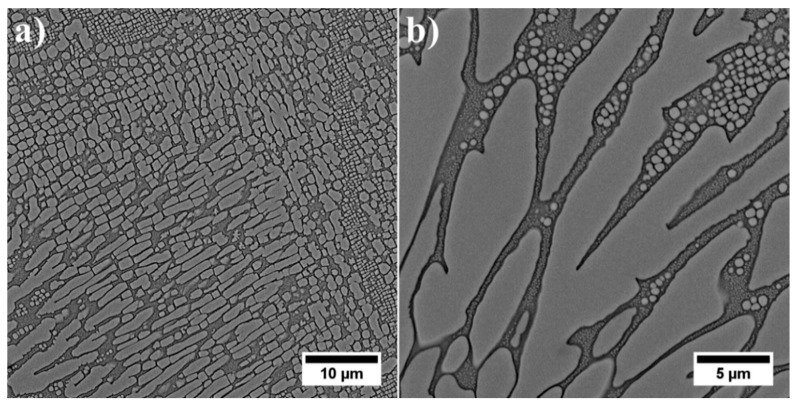
Morphology of γ′ precipitates: (**a**) interdendritic spaces; (**b**) detailed morphology of primary γ′ precipitates in interdendritic spaces.

Much greater variation in local distribution and morphology of all: primary, secondary and tertiary γ′ precipitates in the interdendritic spaces prevented a quantitative analysis and effective comparison with dendritic regions (Figure 4a). The large contribution of eutectic γ-γ′ islands in the microstructure indicated that the liquid phase was strongly enriched in Al in the final stage of superalloy solidification. The complete dissolution of eutectic γ-γ′ islands during solution heat treatment did not take place. The temperature was selected to prevent incipient melting of the superalloy. The primary γ′ precipitates resulting from the L→γ+γ′ phase transformation had a very complex morphology (Figure 4b). The diversity in the morphology of primary γ′ indicated that the L→γ+γ′ transformation took place in a temperature range, so it was monovariant in nature. In the vicinity of eutectic islands there were also fine secondary and tertiary γ′ precipitates.

**Figure 5:**
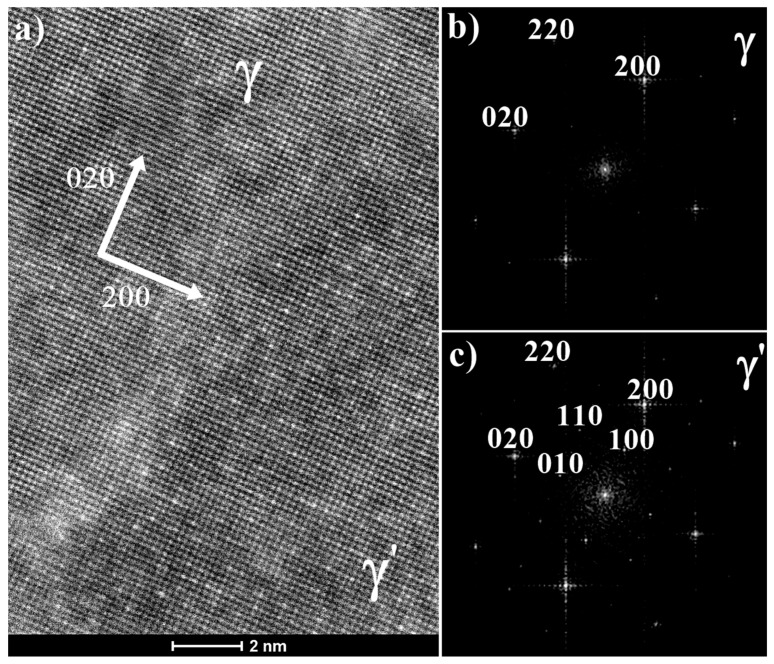
Interface γ/γ′: (**a**) γ and γ′ phases structure in atomic scale resolution, zone axis [001]; (**b**), (**c**) diffraction peaks of γ and γ′ calculated by fast Fourier transformation (FFT).

Moreover, γ/γ′ interfaces for secondary and tertiary precipitates were also investigated at atomic level using the HRTEM technique. Images were recorded with an incident beam parallel to the <001> crystallographic orientation. The HRTEM image revealed interplanar distances to be similar both for γ matrix and coherent γ′ precipitates (Figure 5a). Separate diffraction peaks for γ and γ′ phases were obtain by fast Fourier transformation of nanostructure. For the FCC Ni-matrix phase, the {200}, {220} and {020} reflections revealed that for the ordered L1_2_ structure of the γ′ phase, the {100}, {110} and {010} superlattice reflections are also present. It confirmed that a distinct cube-cube orientation relationship exists between the γ′ precipitates and γ matrix. Crystallographic relationship can be represented by: {100} γ//{100} γ′; <010> γ//<010> γ′, which is referred to as the cube-cube orientation relationship.

**Figure 6:**
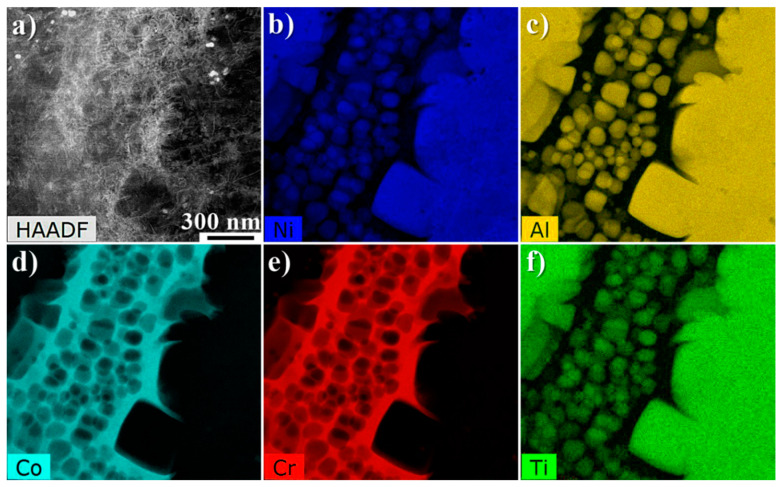
STEM-EDX mapping comprising both dendritic regions and interdendritic spaces: (**a**) selected region for the STEM-EDX mapping; (**b**–**f**) distribution of selected alloying elements in the γ matrix and γ′ precipitates.

**Figure 7:**
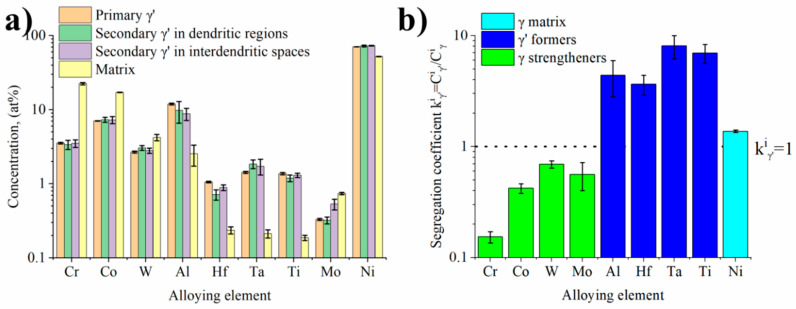
Results of quantitative STEM-EDX analysis of γ′ precipitates and γ matrix: (**a**) chemical composition of the γ′ precipitates and γ matrix; (**b**) segregation coefficient k^i^_γ′_: the calculation based on the concentration of alloying elements.

Figure 6 presents distribution of selected alloying elements in the area, which includes the matrix, secondary and fine tertiary γ′ precipitates. The denuded zones (precipitates-free) were also revealed in the close vicinity of large secondary γ′ precipitates. The fine tertiary γ′ precipitates had a morphology close to spherical. Their mean area was 0.010 μm^2^ (±0.005 μm^2^) and mean perimeter 0.359 μm (±0.101 μm). The size of the precipitates, defined as the equivalent length of the square’s side of γ′ precipitates, was 0.095 μm (±0.026 μm). Shape coefficient factor ζ indicated that this value is much closer to unity namely, 0.877 (±0.069). Distribution maps revealed strong segregation of alloying elements, therefore, they can be clearly divided into γ′ formers and γ strengtheners. In order to get more accurate statistics about difference in chemical composition of precipitates and matrix, a quantitative analysis in several regions of the superalloy was carried out. The concentration of alloying elements in secondary γ′ precipitates in the dendritic regions and interdendritic spaces, primary γ′ precipitates and the γ matrix was determined by STEM-EDX (Figure 7a). No results from tertiary γ′ nanoparticles are presented, due to the overlapping from precipitates and matrix. 

The main alloying elements in the γ′ precipitates were Al, Hf, Ta, and Ti. Their total content in primary γ′ precipitates was 15.8% (±0.4%), in secondary γ′ precipitates in dendritic regions 13.5% (±2.7%) and in the secondary, γ′ precipitates in interdendritic spaces 12.7% (±1.3%). Aluminium dominated, whose concentration depending on the type of precipitate, was in the range 8.8–11.9%. On the other hand, the matrix composition was dominated by Ni, Cr and Co. On the basis of these values, the irregular distribution of all alloying elements between γ and γ′ phases was revelaed. Figure 7b shows the segregation coefficient k, calculated for the alloying elements. The data include primary γ′, secondary γ′ and matrix. It has been noticed that Cr, Co, Mo and W had all k^i^_γ′_ values lower than 1, hence, they enriched the γ matrix channels. Chromium was the most segregated between the two phases, while values close to 1 were found for W and Ni, which indicates a relatively even distribution in the volume. The atomic radius of Ni is 1.49 Å, while for the strengtheners, the radii are bigger: for Co, the atomic radius is 1.52 Å, for Cr: 1.66 Å, for Mo: 1.90 Å, and for W: 1.93 Å [36]. The solid solution elements Cr, W and Mo strengthen the γ phase by increasing the solidus temperature and decreasing the stacking fault energy, which, in turn, influences the thermodynamic stability and the resistance towards dislocation movement [1]. The intermetallic γ′ phase was enriched in elements characterised by the k^i^_γ_ factor above 1, namely Ta, Ti, Al and Hf, for which mean k^i^_γ_ strongly exceeded unity, being 3.64–8.09. Alloying elements in the γʹ phase alter the formation energies of antiphase boundaries, superlattice intrinsic stacking faults and complex stacking faults, and may also directly influence the strength and plasticity of the γʹ phase [37,38].

### 3.2. Dissolution of γ′ Precipitates Studied by in Situ X-Ray Diffractometry 

**Figure 8:**
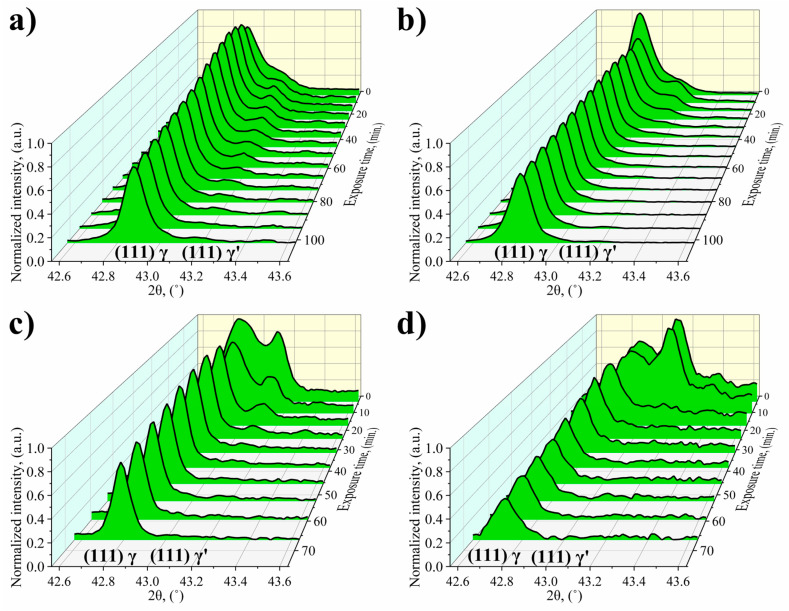
Normalised (111) peak shape evolution during exposure at: (**a**) 1100 °C; (**b**) 1150 °C; (**c**) 1200 °C; (**d**) 1250 °C.

To investigate time-resolved processes, narrow 2ϴ ranges were measured around the peaks of (111) planes, selected due to their high intensity, which was measured in the relatively short reading time for the apparatus used. Figure 8 shows the measured diffraction patterns as a function of 2ϴ and the temperature exposure time. Within the range shown, well-resolved split peaks belonging to the γ and γ′ phases were at approximate positions of 42.9° and 43.1°. At these high temperatures, the intermetallic precipitates partially dissolved, which was indicated by the loss and later disappearance of the peak intensity of the intermetallic phase. It is to be expected that the smaller tertiary particles dissolve first and faster than the larger, they might even dissolve before reaching 1100 °C. A significant feature of the γ′ phase is that the dissolution in the matrix does not proceed by a continuous thinning of each precipitate, but is a more sequential process. Local stability related to elastic interactions affects the selection of the γ′ precipitates to dissolve first, whereas the elastic field results from the misfit parameter. Grosdidier [39], indicate that the dissolution of γ′ precipitates is determined by elastic strains. It takes place through the consecutive “disappearance” of clusters becoming thermodynamically unstable compared to the rest. If the elastic strain field is sufficiently low, the dissolution of precipitates can be much more uniform. It is assumed that the γ′ precipitates are ordered up to their solvus temperature and that dissolution in the surrounding matrix is accompanied by migration of the γ/γ′ interface. Royer [40] noted in AM1 superalloy a partial loss of ordering of γ′ phase at the γ/γ′ interface above 800 °C, and proposed two potential mechanisms for this phenomenon. The first considers disordering of γ′ in the vicinity of the interface induced by the diffusion. The layer of disordered γ′ phase appears at the interface, and its thickness becomes larger with increasing temperature, leading to a drop in the volume fraction of the ordered γ′ phase. The transformation γ′→γ does not occur, due to a lack of chemical composition changes. The second possibility takes into account the fact that the order→disorder transition is initiated by internal stresses at the interface. The temperature evolution of the misfit parameter, which causes an increase of internal stresses, would be at the origin of the increase of the disordering. Similar conclusions were reached by Stepanova [41] in the study of the ZhS 26 superalloy, who pointed out that ordered γ′ precipitates were surrounded by the γ phase enriched in γ′ formers created during the order→disorder transition. In contrast to interface-controlled dissolution, the concentration of alloying elements at the γ/γ′ interface can be commensurate with near-thermodynamic equilibrium. Therefore, an additional metastable γ solid solution is formed, with a composition similar to that of the precipitates, and can coexist in the system for a long time.

**Figure 9:**
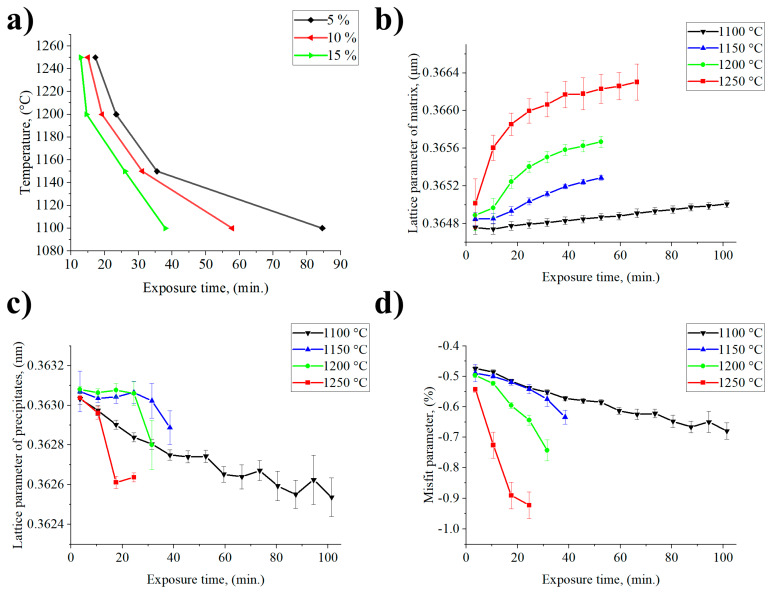
Influence of exposure time and temperature on: (**a**) time-temperature-transformation diagram; (**b**) lattice parameter of γ phase; (**c**) lattice parameter of γ′ phase; (**d**) misfit parameter (δ).

Figure 9 shows the quantitative data obtained by fitting the collected diffraction patterns. By plotting the volume fraction of the precipitates as a function of the temperature over the exposure time, the curves in Figure 9a were obtained. Obviously, initial volume fraction is directly related to the content of γ′ formers. In the case of René 108 the sum of concentrations of Al and Ti was 7.11 wt %. Based on the XRD measurements, the volume fraction of γ′ at room temperature was around 63%. It can be assumed from the shape of the curves that the solution window for the René 108 was in the temperature range 1150–1250 °C. So, the time required to obtain 5% volume fraction of γ′, depending on the temperature of annealing, was between 17 and 36 min. Calculation of lattice parameter of γ and γ′ at room temperature was presented in [19]. During exposure to the high temperature loss of coherency released, the strain in the matrix and the lattice parameter started to increase, depending on the dissolved volume fraction of the γ′ precipitates (Figure 9b,c). It should be noted that the matrix lattice parameter grew with time also, due to local changes of chemical composition, dissolution of other constituents and higher thermal coefficient expansion than for precipitates. At 1100 °C, only a very small increase in the matrix lattice parameter can be observed, which increased at a constant rate. Through the first 17 and 10 min, respectively, for 1100 °C and 1150 °C, the lattice parameter of matrix was constant. At higher temperatures, 1200 °C and 1250 °C, the increase of lattice parameter after 10 min was much more clear, which can correspond both with thermal expansion of the unit cell and enrichment in alloying elements originating from the precipitates. Segregation coefficient calculated on the basis STEM-EDX, revealed high concentration of Al, Ti, Ta and Hf in γ′ precipitates, therefore all these elements enriched the matrix during dissolution. Atomic radii of Ti, Ta and Hf (1.76 Å, 2.00 Å and 2.08 Å, respectively) are higher than of Ni (1.49 Å), which led to an increase of γ lattice parameter [36]. Only Al has a lower atomic radii (1.18 Å). During heating and subsequent holding at the high temperature the solubility of these elements in the matrix increases, regarding the values in Table 1. STEM-EDX revealed that at room temperature the concentration of Al in the matrix channels was 2.5%, while of Ti, Ta and Hf around 0.2% (Figure 7a). Their maximum solubility in the Ni matrix at all the investigated temperatures, calculated by thermodynamic simulation, was much higher.

The γ′ phase was detected in X-ray diffractograms even at the end of the thermal exposure, which means that the particles had not fully dissolved and thus the matrix was still strained by the lattice misfit. For temperatures of 1150–1250 °C, the diffraction peak of the intermetallic phase was not evaluable after certain times of exposure. During heating at 1150 °C and 1200 °C through the first 24.5 min the pronounced plateau of the γ′ phase lattice parameter was observed. The decrease of the volume fraction could indicate that the dissolution process has proceeded without a change of chemical composition of precipitates. The possible origin of this plateau can be the transition from elastic accommodation of internal stresses to plastic relaxation. The source of these internal stresses at interfaces γ/γ′ can be the misfit parameter. Lattice parameters of matrix and precipitates were used to calculate this parameter according to the equation $\delta =\frac{2\left(a_{\gamma \prime }-a_{\gamma }\right)}{a_{\gamma \prime }+a_{\gamma }}$, where a_γ_ and a_γ′_ are lattice parameters of the γ and γʹ phases, respectively. The dependence of the misfit parameter on the time of temperature exposure has an increasing trend at the studied temperatures (Figure 9d). Then, the γ′ phase lattice parameter changed only slightly with time, whereas the lattice parameter of the matrix increased with temperature and rapidly with exposure time. An exception was at 1100 °C, when the lattice parameter of the matrix grew quite slowly, and the relative change at the beginning and after last measurement ∆a_γ_ was 2.50 × 10^−4^ nm. For the temperatures 1150 °C, 1200 °C and 1250 °C ∆a_γ_ was 4.35 × 10^−4^, 7.81 × 10^−4^ and 12.90 × 10^−4^ nm, respectively. Increase in the misfit parameters was closely related to the dissolution of the precipitates, change of chemical composition and probably the loss of a coherent interface. Too high misfit value also has a negative effect on the mechanical properties, because it increases the coarsening rate of the γ′ precipitates [8].

## 4. Summary

René 108 Ni-based superalloy in the initial condition was strengthened by a high-volume fraction (around 63%) of γ′ precipitates, characterised by various morphologies (from the cubic and near-cubic up to spherical). Energy dispersive X-ray spectroscopy revealed that the precipitates were enriched in Al, Ti, Ta and Hf, whereas the γ matrix was stabilised by Cr, W, Co and Mo. An especially high segregation was observed for Ta and Cr, for which the calculated segregation coefficients were 8.09 and 0.15, respectively. In situ XRD experiments showed that the temperature and time of exposure had a significant influence on the kinetics of γ′ dissolution. During the high temperature tests, the volume fraction of precipitates dropped down even by up to 5%, which may indicate that the matrix was strongly enriched in high atomic radius elements Ta and Hf. Time required to obtain such small volume fraction was in the range of 17–85 min for the studied temperatures. Taking into account the decreasing volume fraction of precipitates and accompanying changes of morphology, it can be concluded that their initial stereological parameters and chemical composition strongly influence the change in the lattice parameter, for both γ and γ′ phases, and consequently also the misfit. As a result of precipitates’ dissolution, thermal expansion and the change of local chemical composition, the lattice parameter of the matrix increases with temperature and time. The increasing difference between the lattice parameter of γ′ precipitates and γ matrix led to raised misfit stresses on the interface, which probably favour the order→disorder transition, and then, in consequence, a more intensive dissolution process. The presented approach of the analysis of the kinetics of γ′ dissolution can be helpful in the choice of the solution treatment temperature, obligatory in the fabrication Ni-based superalloy components.

## Figures and Tables

**Table 1:** Change of equilibrium solubility of selected γ′ formers in Ni matrix with increasing temperature calculated by Thermo-Calc software.

Element	1100 °C		1150 °C		1200 °C		1250 °C	
	wt.%	at.%	wt.%	at.%	wt.%	at.%	wt.%	at.%
**Al**	8.57	16.94	9.0	17.71	9.38	18.38	9.85	19.20
**Ti**	10.79	12.92	11.38	13.60	11.98	14.30	12.65	15.08
**Ta**	17.08	6.27	18.52	6.87	20.19	7.58	22.28	8.51
**Hf**	2.58	0.86	3.24	1.09	3.67	1.24	2.98	1.00
